# Book Review

**Published:** 1904-12

**Authors:** 


**The Meaning of a Modern Hospital.** By W. BRUCE CLARKE, M.A., M.B. Pp. 47. London: Longmans, Green & Co. 1904.—At the present time, when hospital construction is as bad as it ever has been, and, we trust, worse than it ever can be in the future, this short pamphlet, written by a surgeon of experience, whom necessity has converted into an expert, will be read with considerable interest. Depleted exchequers, suggestions of wider spheres of usefulness and other causes have combined to drive some of the great London hospitals to wondrous schemes for removal and rebuilding; and among others the author's hospital has had to consider its condition and position, with the result that Mr. Bruce Clarke has not studied the question from the dilettante point of view of a surgical amateur, but has had to acquire information and formulate ideas which would stand the test of practical application. Thus being in the position of one who has worked for years in hospitals, and running the risk of having to work in one of his own planning, fond theories and fads are given no place in his schemes. In dealing with the "Ward Unit," timely stress is laid on the fact that the cubic space allowed per bed must be calculated on a minimum superficial area, that height in a ward will not atone for want of floor space. Windows far above the patients' heads are very properly ridiculed as means of ventilation, and a practical point is made in insisting that a window should divide every two beds. As regards the single story pavilion system of building hospitals he says:—" By improved structural arrangements, judicious attention to sanitary details, and modern antisepsis and asepsis, septic diseases have practically been abolished in almost all hospitals, and it may therefore be regarded as reasonably permissible to construct higher buildings than would have been thought advisable a few years back, providing the height of any pavilion is not such that it will interfere to any considerable extent with the air and sunlight of its neighbours." Five floors he looks upon as probably a working limit. In dealing with isolation accommodation for infectious diseases and septic surgical cases, ten beds per cent. of the whole is suggested as a fair proportion. He is here probably below the mark of useful working as regards septic surgical cases, though above the proportion usually allotted. The value of the surgical work done in a hospital can be made greater by increasing the accommodation for what, for want of a better term, may be called "Conservative Surgery." Of ventilation suffice it to say that artificial systems are said to have been tried and failed, and for this reason:—"The fouling of the air of a building or hospital depends largely on the human beings who inhabit these places, and by far the largest portion of this uncleanliness is derived from the human breath. But few bacteria probably are emitted during quiet conversation—it is mainly during coughing, sneezing, and flights of oratory that a bacterial bombardment occurs." And yet modern surgery does not always cultivate silence on the part of the operator as a virtue. Silence is not only golden, it is aseptic. The conclusion is not hopeful. With the jerry builder at large, backed up by the opinion of certain experts that a modern ward should only be constructed to last fifty years, nothing is more sure than that the hospitals of to-day bid fair to be worse than the vilest efforts of our forefathers, and to become the laughing-stock of our grandsons, if the buildings cling together even so long.

**Practical Physiological Chemistry.** By J. A. Milroy, M.A., M.D., and T. H. Milroy, M.D. Pp. viii., 201 (interleaved). Edinburgh: William Green & Sons. 1904.—An excellent handbook for students in the laboratory, well-arranged, concise and intelligible, is this which has been provided by the Professor and the Demonstrator of Physiology at Queen's College, Belfast. Its brevity is not the result of undue condensation such as is now the fashion in most books compiled for the reading of students, but of the essentially practical nature of the work. The method alone is considered, and for the theory the student is advised to consult his other authorities. Thus an admirable volume has been produced; experiments are fully described and alternative tests are dealt with at some length, while numerous cross references in the text add greatly to the simplicity and coherence of the subject. The chapters on the chemistry of the urine are particularly good, and should recommend themselves not only to the junior students for whom the book is intended. Garrod's methods of separating the pigments are carefully described, and the quantitative analyses are as simple as complicated processes can be made. No space is wasted on the pathological significance of the various abnormal constituents of the urine, and the advantage of this course becomes apparent on comparing the book as a whole with others that profess to enlighten and serve only to mislead. On the whole the authors may be congratulated on their moderation, which, by keeping strictly to the limits laid down by themselves, has produced a not only lucid guide for the beginner, but a valuable work of reference in laboratory methods to the advanced and the expert.

**Medical Tuberculosis: its Rational and Natural Cure, its Several Stages, and Relationship to Cancer.** By R. B. Searle. Pp. 40. London: The Scientific Press Ltd. 1904.—This treatise, written by the Mayor of Dartmouth, is dedicated to philanthropists of all nations, as it is on behalf of suffering humanity dwelling in all climes. It is a tirade against things in general, London in particular, and the fashionable doctor who is considered to be unpractical. The author believes that the typhoid bacillus is antagonistic to that of tubercle, and that anti-typhoid serum should cure the consumptives in our sanatoria; he further believes that pure air is as inimical to the propagation of cancer as of tubercle, and that the germs of cancer might be stamped out by inoculation with anti-toxic serum commenced in infancy. Many facts are needed to give even a semblance of verification to the eccentric theories and views here propounded: the fashionable doctor's "book learning and lecturing" are not yet supplanted by unexperimental speculations and vague fancies.

**Manual of Operative Surgery.** By H. J. Waring, M.S., M.B. Second Edition. Pp. xxviii., 659. Edinburgh: Young J. Pentland. 1904.—The second edition of this manual has been considerably altered from the first. There is a difference in the type, so that although the number of pages is practically the same, the amount of material it contains is much greater. It is also bound into a smaller and more compact volume. At the commencement there is an excellent chapter on The Preparation of an Operation Room and the Sterilisation of all the Apparatus to be used. The chapter on Operations on the Stomach and Intestines has been greatly amplified; a section on The Radical Cure of Femoral Hernia has been added to that on Hernia, one on The Surgery of the Ureters to that on The Urinary System, and much new material, especially on The Operative Treatment of Extra-Uterine Gestation to that on The Female Genital Organs. In fact the whole book has been thoroughly revised and brought well up to date, and may be looked upon as a very useful and compact manual of operative surgery.

**Cleft Palate and Hare-lip.** *The Earlier Operation on the Palate.* By Edmund Owen, M.B., F.R.C.S. Pp. viii., 111. London: Baillière, Tindall and Cox. 1904.—The reader will find in this little book a thoroughly practical description of certain operations for cleft palate and of the author's operation for hare-lip. For the former condition the operation devised by Dr. Brophy, of Chicago, is specially recommended. In this, which is performed in early infancy, the cleft is narrowed by the forcible approximation of the maxillary and palate bones. For three or four weeks the approximation is maintained by wire sutures passed through the maxillæ above the palatal processes and fastened over small leaden plates on the external surfaces of the maxillæ beneath the cheeks. Mr. Owen writes that of late years he has cordially adopted this procedure, although it is attended with increased risk. The first three months of life is considered to be the best age-period for the closure of a cleft-palate, if the health of the child is good. In addition to describing operations, the author discusses well the preliminary preparation of the patients and the after-treatment. The suggestions as to the best ways of overcoming the difficulties of the operations and of the subsequent treatment are excellent. We are of opinion that the book will prove distinctly useful to young surgeons when undertaking their earlier operations for congenital defects of the palate and lip.

**The Care and Feeding of Children.** By L. EMMETT HOLT, M.D., with an introduction by ERIC PRITCHARD, M.A., M.D. Pp. 149. London: Sidney Appleton. 1904.—This admirable little book contains the substance of Dr. Emmett Holt's well-known principles in the care and management of infants and young children. It is arranged in the form of question and answer, and is primarily intended as a manual for children's nurses. Though the style is simple, and as far as possible technical terms are avoided, we doubt whether it is quite within the grasp of the average nursemaid in this country; on the other hand, the trained nurse will find it most useful, and even the medical student might read it with great advantage. Naturally, the modification of milk, according to Dr. Holt's system, occupies a large place. Opinions may vary as to the necessity in most cases of the extreme dilutions he recommends for young infants, for although at the fifth month his formula gives a percentage almost identical with plain milk (but containing more sugar and rather less proteid), he does not allow pure cow's milk to be given till well on in the first year. The question as to whether boiled milk is less digestible than unboiled is answered in the affirmative, although many authorities consider it still undecided, and Pasteurisation is strongly advocated in place of sterilisation. Dr. Eric Pritchard's introduction is mainly laudatory in character, and considering Dr. Holt's reputation among all who are interested in pædiatrics, is a trifle superfluous: on the other hand, a few explanations of words the significance of which is not commonly known on this side of the Atlantic would have been useful. "Rare," for instance, as meaning "raw or underdone," "snug" for "tight," "desserts" for

"sweet puddings," "crackers" for "biscuits," might puzzle many people in this country. Moreover, the substitution of English for American manufacturers of small pieces of apparatus referred to in the notes would be a decided convenience to English readers.

**Insanity in Every-day Practice.** By E. G. YOUNGER. Pp. viii., 110. London: Baillière, Tindall and Cox. 1904.—The author has sought in this small work to give the main outlines of diagnosis and prognosis in the ordinary forms of insanity, and of legal enactments bearing on the insane, and to treat the subject generally from a practical standpoint, that ready assistance may be forthcoming to practitioners who have not had special training in this branch of medicine. The book is written in an easy conversational style, which at times has resulted in some indefiniteness of expression; we are sure that the author's meaning is not always clearly conveyed. We should hardly care to accept in the strict sense passages such as "A delusion is a false belief in some fact;" "Hallucinations . . . become fixed delusions;" "Delusional insanity is . . . characterised by fixed and systematised delusions . . . and is not ordinarily accompanied by any failure of the reasoning faculties;" "G.P. used to be rare in women, but is now becoming commoner, the ratio being about 1 in 4." In writing of general paresis, the author says, "Cases of melancholic type are not unknown." It might have been said they are not uncommon; still more common being cases in which dementia is so early and rapid that the emotional features of G.P. are hardly seen. We object to the insanity of adolescence receiving the alternative title of "Dementia Precox," nor can we agree to the statement that "it is occasionally ushered in by excitement, but more often the patient becomes depressed." Although symptoms of exalted self-feeling are here relatively less frequent in males than females, yet the type of adolescent insanity is rather mania proper than melancholia. When complicated by excessive masturbatic habits, depression may constitute the prevailing emotional tone, but as the author takes the trouble to describe masturbatic insanity as a separate type, there appears no reason to merge its symptoms into those of adolescent insanity. In describing the latter again, he writes, "The tendency of the disease is in all cases towards a slowly increasing dementia. . . . Many varieties of dementia precox have been described . . . but in all of them the dementia slowly increases." Such a gloomy prognosis can only appertain to adolescent insanity when confounded with dementia precox. We regret to notice that the term "acute dementia" is still employed to designate a recoverable disease; it is illogical and misleading to the student. Nor do we think that acute alcoholic insanity is so good a synonym for delirium tremens as acute alcoholic delirium. The book contains useful information on the legal bearings of insanity and on the examination of alleged lunatics. The description of the usual types of mental disease comprises all the salient symptoms, and presents them in a concise and effective manner. The author's large experience of the insane enables him to give many valuable practical hints in the course of what is, as a whole, a decidedly handy volume for the purpose set forth.

**The Extra Pharmacopœia of Martindale and Westcott.** Revised by W. HARRISON MARTINDALE and W. WYNN WESTCOTT, M.B. Eleventh Edition. Pp. 809. London: H. K. Lewis. 1904.—In spite of the addition of more than three hundred new remedies, drugs, and preparations, and a more detailed account of many useful therapeutic agents, the size and weight of the volume have been decreased by the employment of a finer paper. A new section, entitled "Radiology," gives all recent information on radio-active substances, Röntgen rays, high-frequency current, Finsen lamp, and radiant heat in their application to therapeutics. The bacteriological notes and the section on Antitoxins and Organotherapy have been corrected or rewritten. If the physician could adhere rigidly to the official pharmacopœia there would be no need for this book; but as no one either does or could afford to do this, *The Extra Pharmacopœia* becomes an indispensable companion which should be found on every consulting-room table.

**Clinical Studies in Syphilis.** By ARTHUR H. WARD, F.R.C.S. Pp. iv., 156. London: The Medical Times, Limited. 1904.—In these Clinical Studies the author persistently and ingeniously endeavours to show how the varied phenomena of syphilis can be explained if the view be accepted that the cause of the disease is a slow-growing microbe which produces an irritating toxin. Though bacteriological and chemical researches have not as yet succeeded in demonstrating the existence of such a microbe nor of such a toxin, yet the hypothesis of their existence explains so well the clinical features of the disease that it is difficult not to believe in it. Apart, however, from the striking application of the above hypothesis in these Clinical Studies, we have in them an extremely interesting and instructive account of the signs and symptoms of syphilis, including many of its rarer manifestations, an account obviously based upon large experience and careful observation. In addition the principles of treatment are helpfully discussed and many practical directions for such are given of a kind which receive little or no attention in ordinary surgical text-books. The subject-matter of the book, being of such good quality, might appropriately have appeared in better costume than that provided by small print on small leaves of poor paper.

**Manual of Surgery.** By ALEXIS THOMSON, M.D., and ALEXANDER MILES, M.D. Vol. II. Pp. xiii., 723. Edinburgh: Young J. Pentland. 1904.—The general excellence displayed in the first volume of this work is well maintained in the second on regional surgery. A great feature of the book is the surgical anatomy printed in small type at the commencement of each chapter, this matter being a stumbling-block to the student. The subjects treated are brought well up to date. Especially noticeable are the chapters on diseases of the stomach and pancreas and the various affections resulting from gall-stones. The methods of differentiating the urines and estimating the adequacy of the excretory functions of the kidneys are plainly stated. The chapters on peritonitis, including the pneumococcic and gonococcic varieties, and appendicitis, with indications for operation laid down as clearly as possible, are very good. Schleich's method of infiltration anæsthesia is explained, and Killian's tubes for bronchoscopy are mentioned. These are only a few examples that might be quoted as indicating the up-to-date nature of the work. A further point in its favour is that, notwithstanding its small size, it is very readable. We think it the most useful surgical text-book for students we have recently seen.

**Adenoids.** By WYATT WINGRAVE, M.D. Pp. viii., 128. London: Baillière, Tindall and Cox. 1904.—This number of the Medical Monograph Series contains a good and practical account of adenoids. The earlier chapters deal with the anatomy and pathology of the pharyngeal tonsil, and later chapters with the diagnosis of adenoidal hypertrophy and with the palliative, operative, and post-operative treatment of the affection. In the last chapter, written by Mr. Hotten George, the various methods of inducing anæsthesia for the removal of adenoids are considered. Nitrous oxide is the anæsthetic most favoured for patients above three years of age. Below that age there is a liability to the production of an unpleasant degree of asphyxiation and of embarrassing convulsive movements with this anæsthetic. The chief instrument recommended for the operation is the simple Gottstein curette of the rectangular shape. The addition of a cage with projecting teeth to this instrument, the Delstanche modification, is said to be quite unnecessary. One sweeping movement only is made with the curette, and if any fragments of growth are left they are removed with a special forceps, or, in some cases, with a small ring curette passed through the nostril. The finger is introduced two or three times, viz. before operation, after using the curette, and, if forceps are required, again after their use. In our opinion, nitrous oxide anæsthesia does not lend itself well to the introduction of a tonsil guillotine twice, a forceps and ring curette once each, and the finger three times. We approve the selection of nitrous oxide as the anæsthetic, with the limitations mentioned, but we have rarely found it necessary or advisable to introduce foreign bodies into the naso-pharynx so many times. We would, however, advise the practitioner not specially expert in the performance of the operation to use ether or some other agent which yields longer anæsthesia than nitrous oxide. The recognition of adenoids is usually easy and the operation for their removal simple, though not difficult to mismanage; the evils they cause are varied and serious, and the benefits conferred by their successful removal correspondingly great. This little guide-book well points the way to success in treatment, and we can strongly recommend it to general practitioners, the class of readers for whom it has been specially written.

**A Manual for Students of Massage.** By Mary Anne Ellison. Second Edition. Revised by Gulielma Manley. Pp. xii., 126. London: Baillière, Tindall & Cox, 1904.—This is a very useful book for men and women who are being trained to give massage, and has been brought up to date by the addition of instructions on the process as applied to recent fractures and sprains. It pre-supposes throughout the guidance of a trained teacher, and the greater part of the book is taken up with a simple account of anatomy and physiology, tables of the origin, insertion and action of muscles, and fifty diagrammatic plates of the muscles and bones. Of course a knowledge of the anatomy and physiology of the human body cannot be given in 120 pages of clear type, but as an introduction to larger books, or as a handbook for refreshing one's memory, both the text and the diagrams are admirable. On page 50 there is a curious statement on micturition: "The act is completed by increased urine, which quickens the stream and expels the last drops from the urethra." The chapters on Practical Massage and the Weir Mitchell treatment are very interesting, but a caution should be given as to the danger of massage for varicose veins which are thrombosed. There are, indeed, many shrewd suggestions and cautions in the book, and the teaching is, as a rule, sound and good. If patients with, for instance, kidney disease, take massage without medical advice, they cannot expect the attendant to warn them of its dangers. The author points to the risks of such work in the dark, and thus adds to the value of a carefully-planned book.

**The Pharmacological Action and Therapeutic Uses of the Nitrites and Allied Compounds.** By the late Daniel John Leech, M.D.Lond., D.Sc.Vict., F.R.C.P. Pp. 187, with 28 Plates. Manchester: Sherratt and Hughes. 1902.—This volume consists of the Croonian Lectures for 1893, which have been revised and rearranged by Dr. R. B. Wild, and a lecture added on the relation of pharmacology to therapeutics, which affords a valuable indication of the lines upon which Professor Leech taught, and upon which he conducted his own researches. The author's other papers upon the nitrites have been included

**The Management of Lateral Curvature of the Spine, Stooping, and the Development of the Chest in Phthisis.**—By E. Noble Smith, F.R.C.S.Edin. Pp. vi., 132. London: Smith, Elder and Co. 1904.—The author describes in a clear and simple manner his method of dealing with cases of lateral and posterior curvature of the spine, and a chapter is added on a method for developing the chest in phthisis. In respect to lateral curvature and posterior curvature (stooping), he has discussed the advantages to be derived from scientifically-applied exercises, while he maintains that in most cases the use of a mechanical appliance is also advantageous. The particular splint advocated by the author is one known as Chance's splint, which is in use at the City Orthopædic Hospital, and he objects altogether to the closely-fitting plaster of Paris and poro-plastic felt jackets used by Lewis Sayre and others. He considers that the majority of cases of lateral curvature can be treated successfully without massage, and therefore we should not prescribe this expensive method of treatment unless we see some special reason for so doing. The author does not seem to hold a very high opinion of Swedish exercises, but recommends certain movements of his own, which do not seem to differ in any essential principles from the so-called Swedish movements. No doubt every case must be treated on its own merits, and if Mr. Noble Smith's directions are followed success will result in all the less severe cases of this troublesome, but often too much magnified, deformity.

**Medico-Chirurgical Transactions.** Vol. LXXXVII. London: Longmans, Green & Co. 1904.—One of the most noteworthy papers is that by Dr. G. C. Garratt, entitled "Observations on Metabolism in the Febrile State in Man." It is a very elaborate investigation, the report extending to some 160 pages, the result of steady work during a seven years' residence in the London Fever Hospital. A paper of considerable interest just at the present time is that by Drs. Bardswell and Chapman on "The Economic Value of the Sanatorium Treatment for the Working Classes, based upon after-histories." The general outcome of this investigation is that general hospitals should treat a few really hopeful cases on sanatorium lines; and as regards the after-treatment, "the crux of the social factor seemed to be largely a matter of diets; for if a working man, after defraying expenses of house-rent, clothes, &c., had a sufficiently good margin left with which to buy an adequate diet he usually did well, but not otherwise."

**The After-treatment of Operations.** By P. Lockhart Mummery, F.R.C.S. Second edition. Pp. viii., 240. London: Baillière, Tindall & Cox. 1904.—In many text-books after-treatment does not receive the attention it deserves, and this little manual for practitioners and house-surgeons serves to fill up the gap. It is brief, but its teachings are sound, and it may therefore be safely referred to by those for whom it is intended; and though it contains nothing new, it should prove helpful to many who have not had a large practical experience. Some of the illustrations might be redrawn with advantage.

